# Genetic Variation in *FADS* Genes and Plasma Cholesterol Levels in 2-Year-Old Infants: KOALA Birth Cohort Study

**DOI:** 10.1371/journal.pone.0061671

**Published:** 2013-05-08

**Authors:** Carolina Moltó-Puigmartí, Eugène Jansen, Joachim Heinrich, Marie Standl, Ronald P. Mensink, Jogchum Plat, John Penders, Monique Mommers, Gerard H. Koppelman, Dirkje S. Postma, Carel Thijs

**Affiliations:** 1 Department of Epidemiology, CAPHRI School for Public Health and Primary Care, Maastricht University Medical Centre, Maastricht, The Netherlands; 2 National Institute for Public Health and the Environment, Center for Health Protection, Bilthoven, The Netherlands; 3 Helmholtz Zentrum München, German Research Center for Environmental Health, Institute of Epidemiology I, Neuherberg, Germany; 4 Institute of Medical Informatics, Biometry and Epidemiology, University of Munich, Munich, Germany; 5 Department of Human Biology, Maastricht University Medical Centre, Maastricht, The Netherlands; 6 Department of Medical Microbiology, CAPHRI School for Public Health and Primary Care, Maastricht University Medical Centre, Maastricht, The Netherlands; 7 Department of Pediatric Pulmonology and Pediatric Allergology, Beatrix Children’s Hospital, GRIAC Research Institute, University of Groningen, University Medical Centre Groningen, Groningen, The Netherlands; 8 Department of Pulmonary Medicine and Tuberculosis, GRIAC Research Institute, University of Groningen, University Medical Centre Groningen, Groningen, The Netherlands; John Hopkins Bloomberg School of Public Health, United States of America

## Abstract

**Objective:**

Single nucleotide polymorphisms (SNPs) in genes involved in fatty acid metabolism (*FADS1 FADS2* gene cluster) are associated with plasma lipid levels. We aimed to investigate whether these associations are already present early in life and compare the relative contribution of *FADS* SNPs *vs* traditional (non-genetic) factors as determinants of plasma lipid levels.

**Methods:**

Information on infants’ plasma total cholesterol levels, genotypes of five *FADS* SNPs (rs174545, rs174546, rs174556, rs174561, and rs3834458), anthropometric data, maternal characteristics, and breastfeeding history was available for 521 2-year-old children from the KOALA Birth Cohort Study. For 295 of these 521 children, plasma HDLc and non-HDLc levels were also known. Multivariable linear regression analysis was used to study the associations of genetic and non-genetic determinants with cholesterol levels.

**Results:**

All *FADS* SNPs were significantly associated with total cholesterol levels. Heterozygous and homozygous for the minor allele children had about 4% and 8% lower total cholesterol levels than major allele homozygotes. In addition, homozygous for the minor allele children had about 7% lower HDLc levels. This difference reached significance for the SNPs rs174546 and rs3834458. The associations went in the same direction for non-HDLc, but statistical significance was not reached. The percentage of total variance of total cholesterol levels explained by *FADS* SNPs was relatively low (lower than 3%) but of the same order as that explained by gender and the non-genetic determinants together.

**Conclusions:**

*FADS* SNPs are associated with plasma total cholesterol and HDLc levels in preschool children. This brings a new piece of evidence to explain how blood lipid levels may track from childhood to adulthood. Moreover, the finding that these SNPs explain a similar amount of variance in total cholesterol levels as the non-genetic determinants studied reveals the potential importance of investigating the effects of genetic variations in early life.

## Introduction

Elevated total cholesterol (TC), low-density lipoprotein cholesterol (LDLc), and very-low-density lipoprotein cholesterol levels, and low levels of high-density lipoprotein cholesterol (HDLc) levels early in life play a role in the development of adult atherosclerosis, one of the major risk factors of coronary artery disease [1]. This may be partly explained by the fact that plasma lipid levels track from childhood into adulthood [2]–[4], and correlate with the extent of fatty streaks in early life (early atherosclerotic lesions which may progress to advanced atherosclerotic lesions and coronary artery disease) [1], [5]. Therefore, the understanding and control of determinants of plasma lipid levels in childhood is of utmost importance. With such aim in mind, several studies have identified significant associations between maternal anthropometric characteristics and lifestyle [6], and children’s characteristics such as gender [7]–[9], anthropometric characteristic [7], [9], early diet [8], [10], [11], and breastfeeding [12], with children’s plasma lipid levels. Genetic determinants appear to be relevant as well. At least in adults, many single nucleotide polymorphisms (SNPs), each with modest effects, have been found to explain part of the inter-individual variability in plasma lipid levels [13]–[16].

We and others demonstrated that SNPs in the genes coding for the fatty acid desaturases 5 and 6 (*FADS1 FADS2* gene cluster) are associated with the proportions of the various polyunsaturated fatty acids (PUFAs) in adults’ blood and tissues [17], [18] and in plasma phospholipids of 2-year-old infants [19]. As expected, *FADS* SNPs also showed associations with cardiovascular-related outcomes influenced by PUFAs such as the risk of type 2 diabetes [20], [21], coronary artery disease [22], myocardial infarction [23], and metabolic syndrome [24]. In addition, *FADS* SNPs were found to be associated with intermediate phenotypes such as serum or plasma lipid levels in adults and adolescents [13]–[16], [22], [23], [25]–[34]. Recently, Standl et al. showed that the associations between *FADS* SNPs and blood lipid levels are already present in 10-year-old children [35], which raises the question as to whether such associations can be detected even earlier in life.

In the present study we investigated: 1) whether *FADS* SNPs were associated with TC, HDLc, and non-high-density lipoprotein cholesterol (nHDLc) levels in 2-year old infants, and 2) the contribution of *FADS* SNPs in determining plasma lipid levels compared to traditional (non-genetic) determinants.

## Methods

### Ethics Statement

This study was approved by the Ethics Committee of the Maastricht University/University Hospital Maastricht. All parents gave written informed consent.

**Figure 1:**
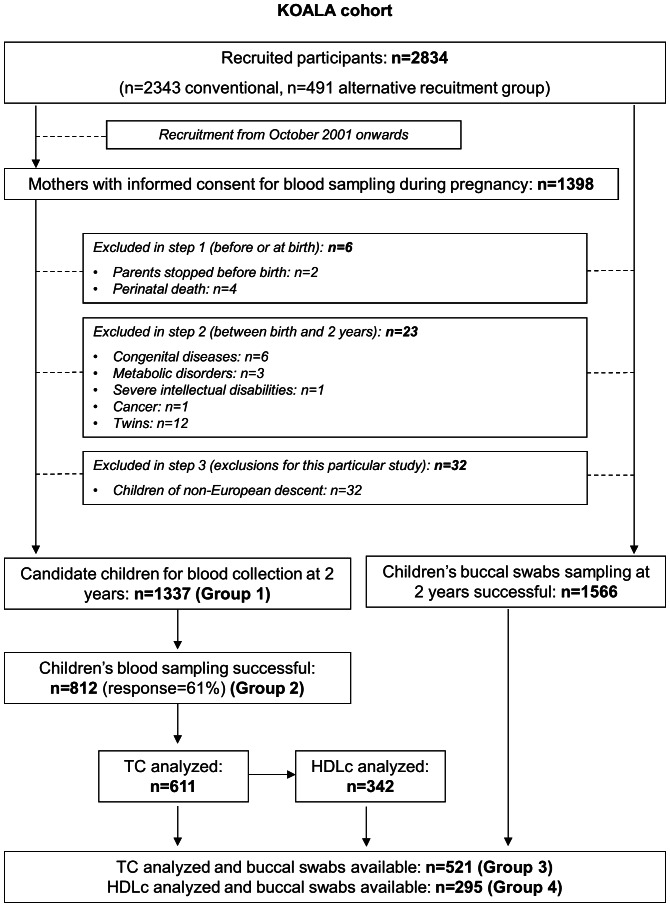
Flow diagram of the study design and participant selection.

CohortThe details of the cohort have been described elsewhere [36]. In summary, the KOALA Birth Cohort Study is a prospective birth cohort in the center and South of the Netherlands. The recruitment of pregnant women started in 2000. Between 2000 and 2002, healthy pregnant women participating in the Pregnancy related Pelvic Girdle Pain Study (PPBS, n = 7526) [37] were invited to participate with their child in the KOALA study. Most women recruited by this means had a conventional lifestyle in terms of diet and child rearing practices. Additional pregnant women with alternative lifestyles were recruited from 2001 to 2002 through organic food shops, anthroposophic doctors and midwives, and Steiner schools. In total, 2834 women were recruited (n = 2343 conventional recruitment group; n = 491 alternative recruitment group) (**figure 1**). Women recruited from 2001 onwards were asked to consent to blood sampling in pregnancy. When children were 2 years of age, parents were asked to consent to children’s collection of buccal swabs and, if maternal consent for blood collection in pregnancy was available, also to children’s blood sampling (eligible children after exclusions, n = 1337). Buccal swabs and blood were successfully collected in 1566 and 812 children, respectively. Due to limited volume of plasma available, TC and HDLc levels were determined in 611 and 342 children, respectively, out of the 812 with blood sampled.

Exclusion criteria were: withdrawal from the study before child’s birth, perinatal death, severe congenital diseases, metabolic disorders, severe intellectual disabilities, cancer, and twins. For this study, children of non-European ancestry were also excluded, as blood lipid metabolism may be affected by ethnicity [38] and the minor allele frequency of *FADS* SNPs differs between ethnic groups [39]. Children’s ancestry was assessed by using information about the country of birth of grandparents, collected through questionnaires filled out by both parents. Children were considered to be of European descent when at least three of their grandparents were born in countries of predominant European ancestry.

The final study population consisted of 521 children with TC analyzed and buccal swabs available, from which 295 children also had HDLc analyzed.

### Plasma Lipids

Non-fasting blood samples were collected in EDTA-tubes by trained nurses, according to a standardized protocol, during a home visit to the child around age 2 years. After centrifugation, the EDTA-plasma was stored in cryovials at −80°C. TC and HDLc were analyzed on an autoanalyser (LX20 Pro, Beckman Coulter, Mijdrecht, The Netherlands) with two kits from Beckman Coulter: *Enzymatic method, nr. CHOL 467825* for TC and *HDLD kit, nr 650207* for HDLc. nHDLc was calculated as the difference between TC and HDLc.

### DNA Isolation and SNP Genotyping

The DNA isolation and SNP selection and genotyping have been described in detail elsewhere [19]. In short, genomic DNA was extracted from buccal swabs using standard methods [40], and afterwards amplified by using REPLI-g UltraFast technology (Qiagen™) as reported before [41]. Five variants of the *FADS1 FADS2* gene cluster (rs174545, rs174546, rs174556, rs174561, and rs3834458) were typed. These variants are associated with the proportions of PUFAs in serum and plasma phospholipids and erythrocytes’ membranes from adults [42]–[44] and children [19]. Additionally, the SNPs rs174545, rs174546, and rs174556 were estimated to tag up to 21 SNPs between basepair positions 61300075 and 61379716 of *FADS1 FADS2*, by using the Tagger tool (http://www.broad.mit.edu/mpg/tagger [45]) with HapMap release 21 data. Genotyping was performed with the iPLEX method (Sequenom, San Diego, CA, USA) by Matrix Assisted Laser Desorption/Ionization Time Of Flight Mass Spectrometry (MALDI-TOF MS, Mass Array; Sequenom).

### Maternal and Children’s Information

Information on potential non-genetic determinants of children’s plasma lipids and possible confounders was retrieved from obstetric reports and questionnaires filled out at different time points during pregnancy and the first two years of children’s life (**table 1**). Maternal and children’s BMIs were calculated as kg/m^2^. In order to standardize children’s BMI for gender and actual age of measurement (which could slightly differ among children), measurements were converted to standard deviation scores (z-scores) using data from the Dutch reference population as the standard [46].

**Table 1:** Maternal and children’s non-genetic potential determinants of blood lipids, possible confounders, and data source.

Maternal information		Source
Determinants	Maternal age at delivery	Q*+obstetric report
	Smoking habits during pregnancy	Q at 14 and 30 wk pregnancy
	Alcohol intake during pregnancy	Q at 14 and 30 wk pregnancy
	Weight and height before pregnancy	Q at 14 and 30 wk pregnancy
	Pregnancy weight gain	Q at 2 wk post-partum
	Parity before index pregnancy	Q at 30 wk pregnancy
Confounders	Maternal education	Q at 14 wk pregnancy
	Recruitment group	Recorded at the time of recruitment
**Children’s information**		**Source**
Determinants	Gender	Q+obstetric report
	Gestational age at delivery	Q at 2 wk post-partum
	Birth weight	Obstetric report
	Weight and height at 2 years	Q at 2 years post-partum
	Breastfeeding duration	Q at 2 wk, 3, 6, 7, 12, and 22 mo post-partum
Confounders	Age at blood collection	Recorded at the time of blood collection

* Q stands for “questionnaire”.

### Statistical Analyses

All analyses were performed with PASW Statistics 18. Agreement of the genotype frequencies with Hardy-Weinberg Equilibrium expectations was tested by chi-square test. Normality of blood lipids was checked by means of histograms and Q-Q-plots.

Associations of genetic and non-genetic determinants with plasma lipid levels were examined in multivariable linear regression models (**table 2**). The associations between *FADS* genotypes and plasma lipids were tested in *model 1* assuming a co-dominant genetic model. Statistical significance was defined by a two-sided alpha level of 5%. Correction for multiple testing was performed by the method proposed by Nyholt [47]. In brief, this method takes into account the correlation pattern between the SNPs and reduces the number of variables in a set to the effective number of variables, which is an estimate of the number of independent tests. We divided the alpha level of 5% by the effective number of independent SNPs (1.26 in our case), yielding a significance threshold of 0.040 required to keep Type I error rate at 5%.

**Table 2:** Variables included in each of the seven examined linear regression models.

Potential determinants of blood lipids included in the models	Regression models nr.*						
	1	2	3	4	5	6	7
*FADS* SNPs	x						x
Gender		x	x	x	x	x	x
Maternal smoking during pregnancy		x	x	x	x	x	x
Maternal alcohol intake during pregnancy		x	x	x	x	x	x
Maternal age at delivery		x	x	x	x	x	x
Pre-pregnancy BMI		x	x	x	x	x	x
Parity before the index pregnancy		x	x	x	x	x	x
Pregnancy weight gain			x	x	x	x	x
Gestational age at delivery				x	x	x	x
Breastfeeding duration					x	x	x
Birth weight						x	x

* Recruitment group and age of blood collection were adjusted for in all models, and maternal education was adjusted for in models 2 to 7.

To test the associations of non-genetic determinants and gender with plasma lipid levels, we started with *model 2* (**table 2**) which included gender, maternal smoking and alcohol intake during pregnancy, maternal age at delivery, prepregnancy BMI, and parity. Subsequently, we built *models 3, 4, 5, and 6* by successively adding the determinants pregnancy weight gain, gestational age at delivery, breastfeeding duration, and birth weight. This allowed us to check the absence of overadjustment bias in the final *model 6.* Such bias could exist when including in a model intermediate variables on a causal pathway from exposure to outcome [48] (e.g. if birth weight would mediate the association between maternal smoking during pregnancy and children’s plasma lipid levels, the inclusion of the former in a model already including maternal smoking could bias the results). Finally, we built *model 7*, including both genetic and non-genetic determinants. As plasma lipid levels may be differently modulated in boys and girls, and some SNPs have been found to exert different effects according to gender [49], the genotype-gender interaction was also tested in this model.

For handling missing data in continuous determinants (**table 3**), we first performed the Little’s Missing Completely at Random test [50], which showed that missing data did not deviate from the “missing completely at random” assumption. Secondly, we imputed missing values through the expectation maximization algorithm using information on maternal age, education, smoking during pregnancy, alcohol intake during pregnancy, gestational age, pre-pregnancy BMI, pregnancy weight gain, birth weight, and child’s gender. Missing values in categorical variables (**table 4**) were coded as a new category. Cases with missing data for SNPs were excluded from the analyses involving that particular SNP.

**Table 3:** Representativeness of the study participants regarding continuous variables.

	Group 1 (n = 1337)*			Group 2 (n = 812)*			Group 3 (n = 521)*			Group 4 (n = 295)*		
	n#	Mean	SD	n#	Mean	SD	n#	Mean	SD	n#	Mean	SD
Maternal age at delivery (years)	1320	32,54	3,80	807	32,80	3,82	516	32,90	3,81	292	32,82	3,62
Gestational age (weeks)	1316	39,7	1,2	805	39,6	1,2	516	39,7	1,2	292	39,7	1,1
Maternal pre-pregnancy height (cm)	1336	170,01	6,37	812	170,01	6,40	521	170,15	6,36	295	170,08	6,02
Maternal pre-pregnancy weight (kg)	1332	67,68	11,65	809	67,95	11,53	519	68,32	11,26	293	68,09	10,59
Maternal weight gain pregnancy (kg)	1241	14,32	5,01	761	14,08	4,79	490	14,10	4,78	281	14,39	4,69
Breastfeeding duration (months)	1327	5,7	4,4	812	5,7	4,4	521	5,9	4,4	295	5,8	4,5
Total cholesterol child (mmol/L)	NA	NA	NA	611	3,80	0,63	521	3,80	0,62	295	3,85	0,62
HDL cholesterol child (mmol/L)	NA	NA	NA	342	1,01	0,22	295	1,01	0,22	295	1,01	0,22
Non-HDL cholesterol child (mmol/L)	NA	NA	NA	342	2,84	0,63	295	2,84	0,63	295	2,84	0,63
Birth weight (g)	1330	3556	476	812	3562	482	521	3579	456	295	3593	469
Weight at 2 years (z-score)	1169	−0,13	0,96	765	−0,16	0,95	497	−0,14	0,94	276	−0,05	0,93
Length at 2 years (z-score)	1161	−0,12	1,07	758	−0,14	1,06	494	−0,08	1,05	277	−0,01	1,02
BMI at 2 years (z-score)	1150	−0,01	1,02	754	−0,04	1,00	491	−0,07	1,02	275	−0,02	0,98

* Groups agree with those defined in Figure 1: group 1 = children candidate for blood collection; group 2 = children with blood sampling successful; group 3 = children with total cholesterol analyzed and genotypes available; group 4 = children with total cholesterol and HDLc analyzed and genotypes available.

# May differ from the total due to missing values.

**Table 4:** Representativeness of the study participants regarding categorical variables.

Variable	Categories	Group 1 (n = 1337)*		Group 2 (n = 812)*		Group 3 (n = 521)*		Group 4 (n = 295)*	
		Frequency	%	Frequency	%	Frequency	%	Frequency	%
Sex	Boys	679	50,8	424	52,2	261	50,1	147	49,8
	Girls	658	49,2	388	47,8	260	49,9	148	50,2
Maternal education	Primary school or lower vocational	41	3,1	27	3,4	19	3,7	8	2,7
	Secondary school or middle vocational	561	42,9	337	42,1	208	40,5	132	45,4
	Higher vocational, or university degreeor higher	707	54,0	437	54,6	287	55,8	151	51,9
	Missings	28	–	11	–	7	–	4	–
Maternalsmoking at any	No	1244	94,4	764	94,9	497	96,7	280	96,6
time during pregnancy	Yes	74	5,6	41	5,1	17	3,3	10	3,4
	Missings	19	–	7	–	7	–	5	–
Alcohol intake during	No	1072	81,2	654	81,0	431	83,5	238	81,5
pregnancy	Yes	248	18,8	153	19,0	85	16,5	54	18,5
	Missings	17	–	5	–	5	–	3	–
Parity before index	0	545	41,3	311	38,5	199	38,6	109	37,3
Pregnancy	1	562	42,6	366	45,4	232	45,0	133	45,5
	≥2	213	16,1	130	16,1	85	16,5	50	17,1
	Missings	17	–	5	–	5	–	3	–

* Groups agree with those defined in Figure 1: group 1 = children candidate for blood collection; group 2 = children with blood sampling successful; group 3 = children with total cholesterol analyzed and genotypes available; group 4 = children with total cholesterol and HDLc analyzed and genotypes available.

## Results

Children’s mean age at blood collection was 25.3 months (range = 22.6–30.6) and did not differ between boys and girls. As shown in **tables 3** **and** **4**, the group with both TC and HDLc analyzed (group 4) virtually did not differ from that with only TC (group 3) with regard to any of the variables relevant for this study. The same was true when comparing group 3 and 4 with group 1 (candidates for blood collection) and 2 (children with successful blood sampling). Plasma lipid levels were normally distributed. Mean values (±SD) for TC, HDLc, and nHDLc of children at 2 years were 3.80 (±0.62), 1.01 (±0.22), and 2.84 (±0.63) mmol/L, respectively.

### Associations between SNPs and Plasma Lipid Levels

SNPs characteristics and genotype and minor allele frequencies are shown in **table 5**. All SNPs were in Hardy Weinberg equilibrium. According to the regression coefficients obtained in *model 1* (**table 6**), heterozygous children (*Mm*) for the SNP rs174545 had about 0.15 mmol/L lower TC than children homozygous for the major allele (*MM*), and homozygous for the minor allele children (*mm*) had 0.30 mmol/L lower TC than *MM* children. These differences were significant even after correction for multiple testing. The results for the other SNPs were highly consistent with those for rs174545, both in terms of magnitude and direction of the association. HDLc was lower in *mm* than in *MM* children (with a difference of about 0.09 mmol/L that reached significance for the SNPs rs174546 and rs3834458). Instead, HDLc levels of *Mm* children did not differ much from those of *MM* children. The possibility that the association between *FADS* SNPs and HDLc levels was better explained by a recessive compared to an additive genetic model was tested and confirmed *post-hoc* by a partial F-test (at α level = 0.05). nHDLc levels were also lower in carriers of the minor allele compared to *MM* children, but the differences did not reach statistical significance.

**Table 5:** Genotype frequencies, minor allele frequencies (MAF), and location of the studied single nucleotide polymorphisms (SNPs).

SNP (major/minor allele)	MM	Mm	mm	MAF (%)	Location
rs174545 (C/G)	233	224	58	33	3′ UTR *FADS1*
rs174546 (C/T)	234	223	59	33	3′ UTR *FADS1*
rs174556 (C/T)	248	220	50	31	Intron *FADS1*
rs174561 (T/C)	251	218	51	31	Intron *FADS1*
rs3834458 (T/del)	233	227	59	33	Intergenic region *FADS1-FADS2*

“MM”, “Mm”, and “mm” stand for homozygous for the major allele, heterozygous, and homozygous for the minor allele children, respectively.

Children with missing data on a specific genotype were excluded from the analyses involving such genotype.

**Table 6:** Associations between *FADS* gene variants and total cholesterol, HDLc, and nHDLc (model 1 according to Table 2).

	Total cholesterol						HDLc						nHDLc					
	n	R^2^ (%)	β	95% CI		p	n	R^2^ (%)	β	95% CI		p	n	R^2^ (%)	β	95% CI		p
**rs174545**	515	2.9					291	1.9					291	1.8				
Mm vs MM			–**.154**	–.267	–.041	.008			–.004	–.057	.050	.893			–.089	–.241	.062	.247
mm vs MM			–**.303**	–.480	–.126	.001			–.092	–.180	–.003	.042			–.198	–.449	.053	.122
**rs174546**	516	2.7					293	1.9					293	1.8				
Mm vs MM			–**.156**	–.269	–.042	.007			–.008	–.062	.045	.760			–.099	–.252	.053	.201
mm vs MM			–**.286**	–.463	–.110	.002			–**.095**	–.183	–.006	.037			–.197	–.450	.056	.127
**rs174556**	518	2.5					294	1.1					294	1.6				
Mm vs MM			–**.142**	–.255	–.029	.014			–.007	–.061	.047	.804			–.068	–.220	.084	.379
mm vs MM			–**.297**	–.486	–.109	.002			–.072	–.167	.023	.136			–.247	–.514	.021	.070
**rs174561**	520	2.4					295	1.1					295	2.0				
Mm vs MM			–**.147**	–.259	–.034	.011			–.011	–.065	.042	.681			–.076	–.228	.076	.326
mm vs MM			–**.279**	–.466	–.093	.003			–.074	–.168	.020	.124			–.251	–.518	.015	.065
**rs3834458**	519	2.6					295	1.8					295	1.7				
Mm vs MM			–**.151**	–.264	–.038	.009			–.006	–.060	.047	.818			–.093	–.244	.059	.231
mm vs MM			–**.289**	–.466	–.112	.001			–**.094**	–.183	.005	.038			–.195	–.448	.057	.129

n: number of children included in the analysis; R^2^: percentage of variance explained (unadjusted R^2^); β: regression coefficient from linear regression analysis indicating the difference in mmol/L between Mm and mm compared to MM (reference category), where MM, Mm, and mm stand for homozygous for the major allele, heterozygous, and homozygous for the minor allele children, respectively; 95% CI: 95% confidence interval; p: p-value. Values in bold are statistically significant after correction for multiple testing (p-value<0.040). Analyses were adjusted for recruitment group and age of children’s blood collection.

### Contribution of Genetic and Non-genetic Determinants

*FADS* genotypes explained 2.4–2.9% of the variance in TC levels, 1.1–1.9% of the variance in HDLc levels, and 1.6–2.0% of the variance in nHDLc (**Table 6** **,** *model 1*). Non-genetic determinants explained 3.5, 4.1, and 10.4% of the variance in TC, HDLc, and nHDLc levels, respectively (**Table 7**, *model 6*). Compared with boys, girls had 0.17 and 0.20 mmol/L higher TC and nHDLc, respectively. Parity was negatively associated with nHDLc, so that children from women with 2 or more children before the index pregnancy had 0.26 mmol/L lower nHDLc than children from primipara women. This association was independent from maternal age. Children in the highest quintile of breastfeeding duration (breastfed for >11 months) had lower nHDLc than those in the lowest quintile (breastfed for ≤1 month). Lastly, birth weight showed a positive association with nHDLc levels. The other determinants tested were not significantly associated with blood lipid levels. The inclusion of BMI z-scores at 2 years instead of birth weight in *model 6* resulted in a positive association between BMI z-score and nHDLc (β = 0.081, 95% CI = 0.003, 0.158), as occurred with birth weight, and in a negative association with HDLc (β = −0.029, 95% CI = −0.057, 0.000).

**Table 7:** Associations between non-genetic maternal and infant’s characteristics and breastfeeding with total cholesterol, HDLc, and nHDLc (model 6 according to Table 2).

	Total cholesterol (n = 521)					HDLc (n = 295)					nHDLc (n = 295)				
	R^2^ (%)	β	95% CI		p	R^2^ (%)	β	95% CI		p	R^2^ (%)	β	95% CI		p
Gender (girl vs boy)	3.5	**.166**	.055	.278	.003	4.1	.008	–.047	.062	.785	10.4	**.204**	.055	.354	.008
Maternal smoking (Y vs N)		.171	–.142	.483	.283		.042	–.105	.189	.576		.244	–.160	.648	.235
Maternal alcohol intake (Y vs N)		–.122	–.276	.033	.123		–.007	–.077	.063	.851		–.002	–.194	.190	.982
Maternal age (years)		.006	–.010	.022	.449		.002	–.005	.010	.537		.021	–.001	.042	.061
Pre-pregnancy BMI (kg/m^2^)		–.002	–.018	.014	.791		.001	–.008	.009	.861		–.004	–.028	.020	.731
Parity before index pregnancy (1 vs 0)		–.092	–.221	.037	.160		–.008	–.069	.053	.798		–.143	–.311	.025	.095
Parity before index pregnancy (≥2 vs 0)		–.060	–.237	.116	.503		–.018	–.102	.066	.674		–**.256**	–.486	–.026	.029
Pregnancy weight gain (kg)		.004	–.009	.016	.541		.002	–.004	.008	.524		–.006	–.023	.010	.441
Gestational age (weeks)		.018	–.035	.071	.496		–.007	–.033	.019	.613		.000	–.071	.071	.999
Breastfeeding duration* (quintile 2 vs1)		–.066	–.244	.112	.467		–.020	–.105	.065	.645		–.212	–.445	.020	.074
Breastfeeding duration (quintile 3 vs1)		–.022	–.192	.148	.799		–.010	–.093	.073	.817		–.202	–.429	.025	.081
Breastfeeding duration (quintile 4 vs1)		–.068	–.248	.113	.463		–.002	–.091	.086	.956		–.052	–.294	.190	.675
Breastfeeding duration (quintile 5 vs1)		–.127	–.322	.068	.202		.047	–.047	.141	.331		–**.362**	–.619	–.104	.006
Birth weight (kg)		.022	–.114	.159	.749		.016	–.049	.081	.627		**.210**	.032	.388	.021

R^2^: percentage of variance explained (unadjusted R^2^); β: regression coefficient from linear regression analysis adjusting for recruitment group, age of children’s blood collection, and maternal education; 95% CI: 95% confidence interval; p: p-value. Values in bold are statistically significant (p-value<0.05).

* Breastfeeding duration was divided into quintiles to correct for its skewed distribution and investigate the presence of dose-effects.

Results from *model 7* (not shown) demonstrated that the regression coefficients for the genetic and non-genetic determinants were virtually the same as when the genetic and non-genetic determinants were tested in two separate models (i.e. *models 1* and *6*, respectively), suggesting that each set of determinants were independent from each other (i.e. no confounding). Moreover, the percentages of variances explained by *model 7* were close to the sum of variances explained by genetic and non-genetic determinants separately (**Table S1**). No significant interaction between genotypes and gender was found.

## Discussion

In the study presented here we show that the associations between *FADS* SNPs genotypes and TC and HDLc levels are already present in 2-year-old preschool children. In addition, we report that *FADS* SNPs and non-genetic factors determine cholesterol levels independently from each other and explain a similar amount of variance in TC levels.

Plasma lipid levels were of the same order as seen in other studies in 2–3 year-old infants, when all expressed in mmol/L [2], [7], [11], [38]. *mm* children had lower TC levels than children homozygous for the major allele (*MM*) (**table 6**). HDLc and nHDLc concentrations were also lower in minor allele carriers than in *MM* children, but statistical significance was not reached in all cases. The fact that the results for all SNPs were very consistent regarding the direction and strength of the associations was expected, based on the relatively high linkage disequilibrium (LD) between the studied SNPs (r^2^≥0.85, D′≥0.99) [19]. The lack of significance for nHDLc could be due to the smaller sample size compared to TC, smaller effect sizes, or higher measurement error (as nHDLc values derive from those of TC and HDLc). Alternatively, it could be that an association with nHDLc is found only when taking diet into account. In this line, Lu *et al.* and Dumont *et al.* found that the association between carrying one or two minor alleles of the *FADS* SNP rs174546 and having lower nHDLc levels became significant only in subjects with high intake of n-3 PUFAs or alpha-linolenic acid, respectively [25], [34]. Unfortunately, we lacked information on infant’s diet and hence could not investigate this possibility.

Expressing the absolute differences in cholesterol levels among genotypes as percentages, heterozygous children (*Mm*) had about 4, 0.5, and 2.5% lower TC, HDLc, and nHDLc levels, respectively, than *MM* children. Differences between *mm* and *MM* children were about 8, 7, and 7% for TC, HDLc, and nHDLc, respectively. This information reveals two interesting points. First, the relative differences between genotype groups seem to be larger in our population of preschool children than in adults or adolescents. Three previous studies presented their data in a way that allowed us to calculate the differences between genotypes as percentage [25], [29], [34]; from these studies, differences between *MM* and *mm* subjects appear to be lower than 4% for either TC, HDLc, and nHDLc. The second observation is that, while the association between *FADS* SNPs and TC seems to agree with an additive genetic model (so that each copy of the minor allele results in an additive decrease in TC, from 4% in *Mm* to 8% in *mm*), the association with HDLc agrees better with a recessive model. In this case, *MM* and *Mm* children have similar levels of HDLc, and levels are lower only in the *mm* group. The hypothesis that may follow this observation and that warrants further research is that, from the three genotype groups, *Mm* subjects may have the lowest TC/HDLc ratio and therefore potentially lower cardiovascular risk.

The biological mechanisms explaining the associations between *FADS* SNPs and cholesterol levels are unknown so far. They are most probably related to the differences in fatty acids proportions in blood and tissues among genotypes. It has been hypothesized that lower percentages of long-chain PUFAs in subjects with the *mm* genotype may explain the lower HDLc levels as a result of lower activation of the peroxisome proliferator activator receptor alpha (which regulates the expression of genes directly involved in HDL production). The lower LDLc observed in other studies, in turn, could be explained by the higher percentages of linoleic acid in *mm* subjects, which could increase membrane fluidity, enhance LDL-receptor recycling, and ultimately lower LDLc levels [29].

Among the non-genetic determinants studied, gender, parity, breastfeeding, and birth weight showed significant associations with plasma cholesterol levels (**table 7**). Girls had higher TC and nHDLc than boys, and children with higher birth weight had higher nHDLc. These results are in line with those reported for preschool children from the ALSPAC Study [7], where girls had higher TC/HDLc ratio than boys, and boys with higher birth weight had higher TC/HDLc ratio at 43 months of age. Although TC/HDLc ratio and nHDLc levels cannot be directly compared, both measures partly reflect LDLc levels. Results on gender agree with studies in older children as well [8], [9]. Women with higher parity (2 or more children before the index pregnancy) had children with lower nHDLc. Rona *et al*. showed an inverse association between parity and TC in 9-year old children [9]. Regarding breastfeeding, we found that longer breastfeeding duration (>11 months) was associated with lower nHDLc levels when compared to shorter duration (≤1 month). A systematic review of observational studies concluded that breastfed infants have higher plasma TC and LDLc levels than their formula-fed counterparts during the first year of life [12]. This may be explained by differences in the composition of breast milk and infant formulas, the former having higher content of cholesterol and saturated fatty acids, but lower PUFAs [51]. However, results obtained in preschool children are mixed: three studies found no association [38], [52], [53] while two studies reported associations with TC levels in opposite directions [7], [11]. Although it is possible that the elevation of cholesterol levels associated with breastfeeding is transient and weakens or vanishes after weaning, more studies are needed to draw definitive conclusions. Lastly, it is worth noticing the positive association between maternal smoking and children’s TC and nHDLc, which is in line with Bekkers *et al.*, who found that 8-year-old children whose mothers smoked during pregnancy had about 0.15 units higher TC/HDLc than children from mothers who did not [6]. The low number of women in our study who smoked during pregnancy (3%) may have limited the power to detect a significant association. Still, the consistency between our results and those by Bekkers *et al*. suggests a true effect of maternal smoking on children’s blood cholesterol levels which warrants confirmation.

A limitation of our study is the lack of infant’s dietary information. Hence, we could not check the contribution of diet to plasma cholesterol levels nor investigate the presence of gene-diet interactions. While interactions between *FADS* SNPs and diet were found in two studies in adults and adolescents [25], [34], no significant interaction was found in a study of 10-year-old children [35].

The studied SNPs explained only a low percentage of variance in plasma lipid level. This was not surprising, considering that several common gene variants discovered through GWAS can also explain, in aggregate, a relatively low percentage of the total variance (about 10%) [13]. Still, it is remarkable that in our study one SNP alone is able to explain as much variance in TC as the non-genetic determinants studied, and to be associated with an 8% difference between genotype groups.

In conclusion, in this study we have shown that *FADS* SNPs are associated with TC and HDLc levels already in infants of 2 years of age, and that the studied SNPs explain about the same amount of variance in TC levels as traditional non-genetic determinants. With this, we provide a new piece of evidence to explain how blood lipid levels may track from childhood to adulthood and to gain insight into the mechanisms that define plasma lipid levels in childhood. We believe that further knowledge on the genetic determinants of plasma lipids will come from the study of other common and rare genetic variants, gene-gene, and gene-environment interactions.

## Supporting Information

Table S1 Comparison of the percentages of explained variance of total cholesterol (TC), HDL cholesterol (HDLc), and non-HDL cholesterol (nHDLc) by genetic and non-genetic determinants.
(DOC)
