# Supplementary material for: Genetic Variation in FADS Genes and Plasma Cholesterol Levels in 2-Year-Old Infants: KOALA Birth Cohort Study
Source: PLoS One. 2013 May 8;8(5):e61671. doi: 10.1371/journal.pone.0061671 (PMC3648514; doi:10.1371/journal.pone.0061671)
Supplement: Table S1 — Comparison of the percentages of explained variance of total cholesterol (TC), HDL cholesterol (HDLc), and non-HDL cholesterol (nHDLc) by genetic and non-genetic determinants. (DOC) [file pone.0061671.s001.doc]

**Table S1.** Comparison of the percentages of explained variance of total cholesterol (TC), HDL cholesterol (HDLc), and non-HDL cholesterol (nHDLc) by genetic and non-genetic determinants.

| **Determinants** | **TC** | | **HDLc** | | **nHDLc** | |
| --- | --- | --- | --- | --- | --- | --- |
|  | n | R^2^ (%) | n | R^2^ (%) | n | R^2^ (%) |
| Non-genetic determinants (model 6, with whole population) | 521 | 3.5 | 295 | 4.1 | 295 | 10.4 |
| Non-genetic determinants (model 6, restricted to children with known rs174545 genotype) | 515 | 3.1 | 291 | 4.0 | 291 | 10.2 |
| rs174545 genotypes (model 1) |  | 2.9 |  | 1.9 |  | 1.8 |
| rs174545 genotypes and non-genetic determinants (model 7) |  | 5.8 |  | 5.9 |  | 10.7 |
| Non-genetic determinants (model 6, restricted to children with known rs174546 genotype) | 516 | 3.5 | 293 | 4.2 | 293 | 10.4 |
| rs174546 genotypes (model 1) |  | 2.7 |  | 1.9 |  | 1.8 |
| rs174546 genotypes and non-genetic determinants (model 7) |  | 6.1 |  | 6.0 |  | 11.0 |
| Non-genetic determinants (model 6, restricted to children with known rs174556 genotype) | 518 | 3.4 | 294 | 4.1 | 294 | 10.4 |
| rs174556 genotypes (model 1) |  | 2.5 |  | 1.1 |  | 1.6 |
| rs174556 genotypes and non-genetic determinants (model 7) |  | 5.8 |  | 5.1 |  | 11.0 |
| Non-genetic determinants (model 6, restricted to children with known rs174561 genotype) | 520 | 3.6 | 295 | 4.1 | 295 | 10.4 |
| rs174561 genotypes (model 1) |  | 2.4 |  | 1.1 |  | 2.0 |
| rs174561 genotypes and non-genetic determinants (model 7) |  | 6.0 |  | 5.1 |  | 11.0 |
| Non-genetic determinants (model 6, restricted to children with known rs3834458 genotype) | 519 | 3.4 | 295 | 4.1 | 295 | 10.4 |
| rs3834458 genotypes (model 1) |  | 2.6 |  | 1.8 |  | 1.7 |
| rs3834458 genotypes and non-genetic determinants (model 7) |  | 5.9 |  | 5.9 |  | 10.9 |

Model 6 includes the following variables: gender, maternal smoking during pregnancy, maternal alcohol intake during pregnancy, maternal age at delivery, pre-pregnancy BMI, parity before the index pregnancy, pregnancy weight gain, gestational age at delivery, breastfeeding duration, and birth weight.

Model 1 includes the following variables: *FADS* genotype.

Model 7 includes the following variables: *FADS* genotype, gender, maternal smoking during pregnancy, maternal alcohol intake during pregnancy, maternal age at delivery, pre-pregnancy BMI, parity before the index pregnancy, pregnancy weight gain, gestational age at delivery, breastfeeding duration, and birth weight.

n: number of children included in the analysis.

R^2^: percentage of variance explained (unadjusted R^2^) derived from linear regression analysis, adjusting for recruitment group and age of children’s blood collection.
